# Children Exposure-Related Behavior Patterns and Risk Perception Associated with Recreational Beach Use

**DOI:** 10.3390/ijerph16152783

**Published:** 2019-08-03

**Authors:** Alesia Ferguson, Courtney Del Donno, Emmanuel Obeng-Gyasi, Kristina Mena, Tanu Kaur Altomare, Rosalía Guerrero, Maribeth Gidley, Larissa Montas, Helena M. Solo-Gabriele

**Affiliations:** 1 Built Environment, North Carolina A&T, Greensboro, NC 27411, USA; 2 Environmental and Occupational Health, University of Arkansas Medical Sciences, Little Rock, AR 72111, USA; 3 School of Public Health, University of Texas, El Paso, TX 79968, USA; 4 School of Public Health, University of Texas, Houston, TX 77030, USA; 5 National Oceanic and Atmospheric Administration (NOAA), Atlantic Oceanographic and Meteorological Laboratory (AOML), Miami, FL 33149, USA; 6 University of Miami, Cooperative Institute for Marine and Atmospheric Studies (CIMAS), Miami, FL 33149, USA; 7 Department of Civil, Architectural, and Environmental Engineering, University of Miami, Coral Gables, FL 33146, USA

**Keywords:** beach surveys, oil spill chemicals, children exposure

## Abstract

Background: Oil spill chemicals (OSCs) result not only from the crude oil components but also from dispersants used in the clean-up activities, where some may result in adverse health effects under certain exposure and dosage conditions. One of the main populations of concern for exposure to OSCs are children, who are frequent beach users. Activities such as ingestion of and digging in sand can increase dermal and ingestion exposure. Longer times spent at the beach can also increase exposures for all routes. Objectives: The Beach Exposure and Child Health Study (BEaCHeS) was initiated to evaluate the risk of exposure to children from oil contaminants. Reported here are results for surveys collected, as a part of the project, to address exposure-related behavior patterns and risk perception for parents or guardians who visit the beach. Methods: Over 400 parental surveys were collected at four beaches, two in Miami and two in Texas, to evaluate children’s exposure related activities. Surveys consisted of three general sections: demographics, exposure, and risk perception. Surveys were analyzed in REDcap and Stata to evaluate demographic and regional differences on activities related to beach behavior and potential exposures to oil contaminants (e.g., how much time spent on beach, cleaning habits following beach activities). The statistical analysis included the mean and standard errors, along with regressions to evaluate associations between parameters. Results: Overall, the data showed high variability in how children play on the beach, influenced more by age and less by gender. Variations were also seen in certain variables by beach region (e.g., hygiene practices). By race, variations were seen in income, distance of travel to beach, and preferred method of communication for beach warning. Other important findings are reflected in the article. Discussion: The data presented here may prove useful for those evaluating children exposures to a variety of contaminants, chemical, or bacterial in origin. In addition, coastal managers may find the risk perception and general behaviors useful for planning and maintenance of beach areas.

## 1. Introduction and Background

During April 2010, the Deepwater Horizon (DWH) oil drilling platform caught fire and exploded in the Gulf of Mexico, spilling over 200 million gallons of crude oil. This disaster led to significant losses in the area’s economy because the oil spread over many miles of coastline and caused the closure of over 80 beaches [1,2]. The large extent of the closures was partly due to the uncertainty of health risks associated with the spill and its effect on populations that use these beach areas. Oil spill chemicals (OSCs) result not only from the crude oil components but also from dispersants used in mitigation, where some may result in adverse health effects under certain exposure and dosage conditions [3,4]. Spills, caused by negligence, structural or mechanical failure, or natural events, are likely in the future. As a result, even after the DWH disaster, there is a likelihood that oil spills will contribute to the presence of potentially hazardous chemicals in areas where humans spend time.

One of the main populations of concern for exposure to OSCs are children. Children are frequent beach users, where families see the beach as not only a form of exercise but a healthy means for relaxation and bonding with nature [5]. However, if beaches are contaminated, children may be at greater risk to exposure due to their unique activity patterns and their toxicological susceptibility. Previous studies have evaluated the unique behaviors of children for a number of exposure scenarios [6,7,8,9,10,11,12]. Their play habits in general tend to put them at higher risk to exposures through the three routes of exposures, ingestion, dermal, and inhalation. For the beach environment, this is also true, where for example children are more likely to ingest contaminated sand and water [13,14]. Children may also potentially have greater dermal risk when they dig and bury themselves in the sand and sit in shallow water, which generally has the highest level of contaminants [15,16]. Children are also closer to the ground, which can result in a greater inhalation risk to any OSCs that have accumulated in the sand or water. Children are also more susceptible to chemical exposures, where toxic chemicals can disrupt and impact the development of their organs and organ systems.

There have been limited studies that address beach activities (e.g., length of stay, number of visits) and individual habits (e.g., digging, wash activities before a meal) that might influence exposures. One potentially useful study is the United States’ Environmental Protection Agency’s National Epidemiological and Environmental Assessment of Recreational Water (NEER) study conducted over a three year period to document behaviors for 29,100 beach visitors in Rhode Island, Alabama, Mississippi, Indiana, Michigan, and Ohio [17,18]. Although this study focused on lake areas and microbial exposure versus chemical exposures, it still offers useful activity patterns for exposures to chemical pollutants found in coastal beach sands. Valuable data gathered included activity patterns across age (i.e., adults and children), sex, race, and ethnicity on certain behaviors (e.g., digging in sand and body buried in sand). The study also addressed the number of visits to the beach, distance traveled to beach, and wash events following play activities on the beach, offering insight on various types of activities, relevant for both acute and chronic-long term potential exposures.

Other studies in the literature offer some general information on the activities of families and children in the beach environment, however the goal of these studies differ. Some of these studies look at human activity at beaches (e.g., length of stay, preference for clothing based on weather, time of year preferred) for coastline management or for tourism prediction and planning [19,20,21,22]. A more recent random survey of individuals visiting 11 beaches—also along the southern coastline area—showed that most people visit the beach with one or more people and that taking children to play at the beach was the second most common reason for going to the beach [23]. In addition, the majority of people drive to the beach, the majority stay 2–4 h or more, and a little less than half visit five or more times per year.

In 2017, this group of researchers were funded through the Gulf of Mexico Research Initiative to evaluate children‘s health risks to oil spill chemicals by integrating realistic play activities and distributions of oil spill chemicals in the beach environment. The project entitled “Beach Exposure and Child Health Study” (BEaCHeS) utilized a health risk paradigm approach that requires information on the temporal and spatial distributions of OSC in the nearshore environment and beach behavior activities across a representative population of children that would allow aggregate health risks estimates based on the three routes of exposure (i.e., inhalation, dermal, ingestion).

The overall project involved a number of data collection tasks and risk modelling steps to evaluate high-risk groups and particular activities and beach profiles or fate and transport dynamics that drive exposure over time periods following a spill event. Other data collection activities included as a part of the BEaCHeS study: videotaping and video-translation to document meso (i.e., frequency of activities in time segments) and micro (frequency and duration by the second) activities, hand presses to assess soil adherence to the palm of hand, and body rinses to measure full body soil adherence.

This paper focuses on information gathered from surveys collected on the project, where parents or guardians of children under the age of six was the focus group. The survey was intended to help capture a broader scale of exposure factors for children to ultimately improve health risk estimates. The survey also included a section on risk perceptions to understand what might influence a families’ use of the beach area and how families prefer to receive warning messages for beach conditions. Past studies have shown that messages from the media on the environmental conditions can heavily influence beach usage as opposed to educational campaigns that use scientific data [24]. Although the study focused on Gulf regions, information gathered from these surveys may be adaptable to other regions and countries.

## 2. Methods

### 2.1. Survey Instruments

No ideal beach survey instrument was found in the literature that would allow for the collection of detailed children’s activity patterns related to exposure to OSCs in the beach environment. Researchers therefore developed a new instrument. The survey instrument was designed to complement other data collection activities on the project in order to gather a broad set of interrelated children activities that influence exposure.

Researchers completed development of the survey after numerous iterations between researchers. The demographic section consisted of 7 questions and sub sections to gather age, race and nationality information, along with occupation, income, and makeup of family. The exposure section, which is considered the most relevant section for computing risk, consisted of 29 questions and subsections to address family and child related exposure patterns. The final risk perception section consisted of 15 questions and subsections to evaluate risk ranking and messaging preference for beach warnings. Institutional review of human subject protocols and consent forms were obtained primarily through the University of Miami (IRB 20140140-MOD00023226). Other IRB approvals were obtained through the University of Texas (IRB #HSC-SPH-18-0396), where the University of Arkansas for Medical Sciences and later University of North Carolina Agricultural and Technical State University fell under University of Miami’s IRB. Participants were compensated with a $25 gift card for completion of the survey. Parking costs were also covered for participants that took part in the video-taping protocol. Inclusion criteria was a parent or guardian with at least one child under the age of six who had taken a child to the beach within the last year.

### 2.2. Recruitment and Data Collection

**Figure 1:**
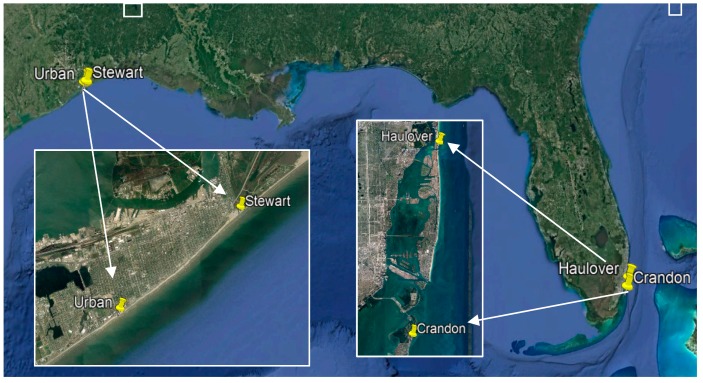
Gulf Beach locations for data collection.

Over the summer months of 2018, researchers walked along the target beaches in Miami, Florida (Crandon (5°42′10″ N, 80°09′14″ W) and Haulover (25°54′03″ N, 80 07′19″ W)) and Galveston, Texas (Stewart (29°18′24″ N, 94°45′56″ W) and Seawall/Urban (29°16′12.4″ N, 94°49′7.4″ W)) (Figure 1). All beaches are public but have varying fees for parking. At the beaches families were approached to encourage them to participate in the survey of children’s beach behaviors. Some surveys were collected at the same time as videotaping during the summer of 2018 and other surveys were collected in the early fall of 2018. Surveys were available in English and Spanish for the family’s convenience. Researchers read a script to potential participants that explained the purpose of the survey, how it would be used, and how their personal information would be protected. Parents and guardians completed consent forms before proceeding with surveys. Although researchers typically approached families on the beach to compete the survey, the first part of the survey verified that the person completing the survey was at least 18 years old and had taken a child to the beach in the last year. If these criteria were satisfied, they were then asked to complete the remainder of survey. Some parents requested help completing surveys (i.e., comprehending the intention of certain sections). Participants were not forced to complete all sections; however, the completed surveys were checked in case participants accidently forgot to answer pages.

### 2.3. Data Refinement and Statistical Analysis

A total of 407 surveys were collected at the four beaches. Completed surveys were uploaded into the online Box Storage Platform and assigned a file name. File names corresponded to beach area and numeric number or a child ID, if the family also participated in the videotaping portion of the study. Surveys were then manually entered into the REDCap (Research Electronic Data Capture) platform. REDCap, was developed at Vanderbilt University in 2004 as a secure data collection or entry tool, with some built in analysis tools [25]. There were 18 surveys completed in Spanish which were identified and translated for entry. Spanish entries were checked by a second researcher on the project fluent in Spanish. After discussions between researchers, 7 surveys were deemed to be incomplete or incorrect and excluded, leaving 400 surveys in the final analysis. Among the 400 surveys, 199 were from the Miami Beaches of Crandon and Haulover, and 201 were from the Texas beaches of Stewart and Urban. Out of the 400 surveys, 80 were collected from parents or guardians whose children (aged under 7 years) also participated in videotaping, soil adherence experiments and other field measurements. These 80 surveys corresponded to responses for 125 children.

Initially basic statistics were performed in REDCap to compute means, medians, and standard deviations for demographic, exposure and risk questions across respondents. Stata SE/15.0 (StataCorp, College Station, TX, USA) was used to perform further analysis with Excel 2016 used to generate figures. To evaluate logical relationships, linear and binary logistic regression were used to determine associations between both continuous and categorical variables in sections of the survey and across sections. Each outcome and predictor variable was explored in individual models in both linear and logistic regression analysis.

For example, associations were evaluated between beach locations and race versus other demographics factors and factors that relate to exposure and risk. Level of significance was identified as *p* < 0.05 with a *p* value of <0.10 considered as moderately significant. Non-response rates (NR) are often reported. High NRs can reflect a question that is either not applicable to a respondent, difficult to answer, accidently missed, or is uncomfortable to answer. High NR rates can affect the ability to confirm significant differences between groups.

## 3. Results and Discussion

The surveys were de-identified before analysis. Responses are presented in three sections: demographics, exposure assessment, and risk perception. Demographics focused on race and ethnicity of the parents, exposure assessment focused on family and individual children’s activity patterns, and risk perception focused on the parent’s perceptions of various risks associated with the beach environment. Associations between variables in all three sections were also explored.

### 3.1. Demographic Results

Results from the demographic section of the survey showed that the mean age of the respondents completing the survey was 37.9 (± 11.5) years old (see Supplementary Table S1). The person taking the children to the beach and completing the survey were parents (79.8%), grandparents (10.5%), or guardian or other (9.8%). Guardian or other included aunts or uncles and older siblings. As a result, the person completing the surveys ranged in age from 17 years to 73 years Most survey respondents were women at 71.8%, while men represented 28.2%. This distribution may be due to women being the primary caregiver, or the designated person in the family to complete the survey. The majority of respondents were White (75.5%), followed by Black or African American (18.4%), and Other (6.1%), where Other included American Indian or Alaska Native (1.4%), Chinese (1.7%), Filipino (0.8%), Vietnamese (0.3%), other Asian (1.4%), and other Pacific Islander (1.1%).

For the 46.6% who identified as Hispanic, they further identified as Mexican including Mexican American and Chicano (13.6%), Cuban (7.9%), Columbian (4.4%), Puerto Rican (3.8%), Venezuelan (2.6%), or another Hispanic, Latino, or Spanish origin (14.1%). For the question on country of birth, there was a high 32.0% no response rate, however the majority of the respondents (69.1%) were born in the United States. Regarding household income, respondents mostly fell in the $51,000–100,000 income bracket (25.4%). This is followed by people who make over $100,001 (19.9%), $30,001–50,000 (16.2%), $20,001–30,000 (11.8%), less than $12,000 (8.6%), and then $12,001–20,000 (5.0%) (NR for this question was 13.1%). There was a general trend of income increasing with age. For example, there was a significant difference in age between those that made less than $12,000 versus those that made between $50,000 to $100,000 (*p* < 0.001). There was also a positive association between income and hours spent at work (*p* = 0.014), even after adjusting for age and gender. There was no association between income and hours worked in the primary job.

Most individuals answered about their primary occupation (NR = 6.8%), where example of occupations included student, housewife, bank teller, and dentist, with an average of 38.3 h per week of work. Only 23 respondents indicated that they had a second occupation, where 16 responded on hours worked with mean of 18.1 h. To the question about occupation of spouse, 60.2% responded. Spouse’s occupations included barber, mechanic and pilot. Only 39.1% of those answered for the hours worked by their spouse with a mean hours worked of 42.8 h.

All respondents indicated that they were interviewed while going to the beach (NR 4.0%), although for some participants who were also a part of the videotaping study, surveys may have been started beforehand at home and collected on the day of the field study at the beach. For location of home, 79.9% answered that their home was found in the state, which means the other individuals were visiting from another state or country.

### 3.2. Exposure Assessment Results

The exposure assessment section of the survey had three main subsections. Subsection 1 included questions that pertained to the family’s beach going habits, and age and sex of children in the home. Subsection 2 pertained to each child’s individual habits. Subsection 3 looked at what items children put in the mouth and like to collect, in addition to sunscreen use and overall hygiene practices.

#### 3.2.1. Exposure Subsection 1

Results show that most of the participants lived within 30 miles of the beach (69.6%), and most drove to the beach (91.3%) (see Supplementary Table S2). For the number of children under 18 identified as living in the home that go to the beach, the answers ranged from 0 to 8 with a mean of 1.85 children. For the number of children they typically take to the beach, the answer ranged from 1 to 14 with a mean of 2.16. The maximum of 14 children indicated by a respondent could potentially be due to a participant bringing their entire family (including older cousins, nieces, and nephews) and children’s friends to the beach or a potential misunderstanding by the participant. In a binary logistic regression to compare responses in Miami versus Texas, those in Texas were more likely to have a higher income, work more hours, live further from the beach, and spend a longer time when at the beach; however, none of these findings were statistically significant.

Parents were allowed to list age and sex for up to seven children who lived with them or that they take to the beach. Across the surveys, 258 participants identified that their first child was under the age of six years (with 48.7% female), 117 participants identified that their second was under the age of six years (with 52.5% female), 30 participants identified that their third child was under the age of six years (with 51.1% female), 9 participants identified that their fourth child was under the age six years (with 50.0% female), and 6 participants identified that their fifth child was under six years (with 36.4% female). No participants identified that their sixth and seventh child was under the age of six years

The next series of questions were designed to evaluate the number of times a child might go the beach, how long they stay and what might influence the number of visits. Based on responses, most families go to the beach during the summer (69.8%) or all year long (29.2%), as might be expected. Some families go during the fall (4.9%), spring (11.0%), winter (1.8%), or other times of year (1.3%) (NR 2.3%). Over 70% of the participants said they go to the beach based on the weather being a nice, sunny day. The other reasons included the children being out of school (61.4%), air temperature (21.6%), water temperature (15.9%), and various other reasons (8.0%), such as free time and for child’s happiness. Along with the weather and seasonal preferences, participants also noted what impacts the time of day they take their children to the beach. The most prominent factor that affects a parent’s or a guardian’s choice for time of day to take their children to the beach would be if the sun was intense enough at 58.4%. Other choices included whether a work/school schedule (48.9%) would allow, if it was hot enough (45.3%), if the traffic was not too bad (27.1%), and other reasons (4.2%) (NR 5.0%).

On average, 39.9% of participants take their children to the beach once a year or more, while 36.3% of people said they take their children to the beach once a month or more. Another 17.1% responded that they take their children as much as once a week or more, and 6.6% were uncertain of the number of the times they take their children (NR 2.3%). There was a difference in how often parents take their children to the beach by region, where those in Miami more often took their children once a week or more, while Texas parents/guardians more often took their children once a year these differences were not significantly different by beach location. Over 46% of people said they stay at the beach for 3 h or longer when they take their children, followed by people staying for 2–3 h (37.2%), 1–2 h (13.0%), uncertain of how long they stay (2.0%), and less than 1 h (1.3%) (NR 2.0%). To compare how long participants stay when they go without children, 39.3% stayed more than 3 h, 29.8% stayed between 2 and 3 h, 13.4% stayed between 1 and 2 h, 9.5% only go with children, and 5.4% were uncertain (NR 2.8%). There was therefore a tendency to stay longer with children. Participants reported that they mostly bring toys (86.1%) and additional friends (45.5%), animals and pets (7.9%), and other items (22.0%) to the beach (NR 4.5%). For this question, participants were allowed to respond to all that applied. Some other items that they brought to the beach included umbrellas, food, shovel, buckets, sunscreen, balls, strollers, blankets, tents, chairs, blankets, coolers, surfboard, kites, and music.

In a linear regression that evaluated associations between income and exposure variables of interest, there was no association between income and frequency of visits to the beach, distance from beach, and length of time at beach. However, in a regression of length of time and variables of interest, length of time at the beach was positively and significantly associated with age (*p* = 0.001), frequency at beach (*p* = 0.015), and moderately significantly associated with distance from beach (*p* = 0.053).

#### 3.2.2. Exposure Subsection 2

The second part of this section focused on individual children’s activities, with a specific interest in children under 6 years of age. Therefore, the survey only allowed responses for activities for the first to fifth child in the family (youngest to oldest). Researchers designed the survey on the assumption that it was unlikely that families had more than 5 children under 6 years of age. Activities were analyzed by age groups, where there were 56 one-year olds, 67 two-year olds, 66 three-year olds, 66 four-year-olds, 82 five-year-olds, and 83 six-year-olds. There were 327 children older than six not included in the analysis below, because this project is focused on younger children. Respondents were able to select 0–100% for any single category of activity or behavior, which could result in total percentages over 100.

**Figure 2:**
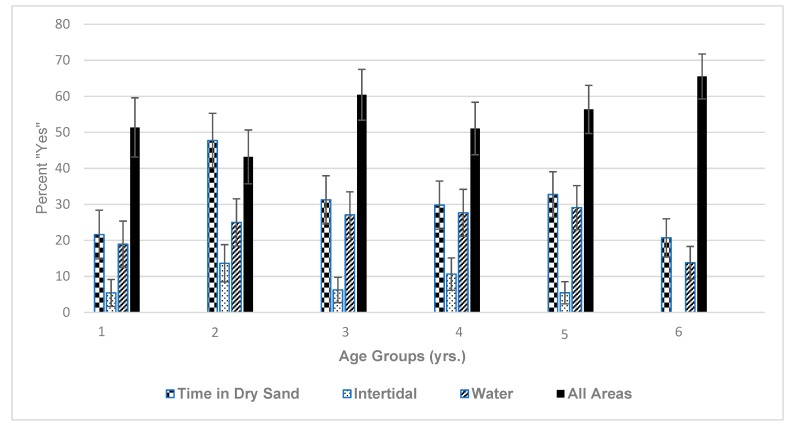
Preference for Playing in Designation Areas by Age Group. For ‘all areas of play: a significant difference between children aged 2 years and 6 years (*p* = 0.022) and a moderately significant difference between 2 years and 3 years (*p* = 0.094). For ‘dry sand play’, there was a significant difference between children aged 1 year and 2 years (*p* = 0.010), for 2 years and 6 years (*p* = 0.003), and a moderately significant difference between 2-year and 4-year-olds (*p* = 0.037). The 1-year-olds and 6-year-olds choose ‘dry sand’ less than other areas of play. For ‘water play’, there was a moderately significant difference between children aged 3 years and 6 years (*p* = 0.091), and between 4 years and 6 years (0.081), while there was a significant difference between children aged 5 year and 6 years (*p* = 0.045).

Researchers were interested in where children like to spend time while at the beach, where the survey choices were ‘dry sand’, ‘water’, ‘intertidal’, and ‘all areas’. The ‘intertidal’ region of the beach is described as the area where the tides routinely wets the sand, in high or low tide [26]. For the children under the age of six, all age groups preferred to play in the ‘all areas’, followed by ‘dry sand’, ‘water’, and then ‘intertidal’ (Figure 2). In general, 2-year-olds choose the ‘all areas of play’ less than other ages groups, but seemed to prefer ‘dry sand play’, more than other groups.

Another related question on the survey addressed the percent time respondents believed their children spent in each location (i.e., ‘dry sand’, ‘intertidal’, ‘water’, ‘other’) (Not shown in any figure below). Percent time spent ranged from 33.6% to 44.5% for ‘dry sand’, from 26.7% to 34.8% for ‘intertidal’, from 31.4% to 42.9% for ‘water’ and 10.0% to 57.5% for ‘other’ for ages 1 year to 6 years Respondents may have interpreted ‘other’ to mean intertidal, rocks or concrete areas of the beach. Overall ‘dry sand’ play was more popular among most age groups, except for the 6-year-olds that spent the most time in ‘water’. There were significant findings for some of these differences.

Younger children are carried to the beach by their parents more often than the older children; therefore, many of their activities such as ‘water’ play are dictated by where the parents want to be. Overall, we see some general trends, where children prefer the dry sand and water as compared to the intertidal area. In addition, older children preferred water as compared to younger children with the younger ones preferring dry sand. In an analysis of females versus male children, there was generally no significant difference in where they like to spend time (i.e., intertidal, water, sand, all areas) across all age groups. However, 5-year-old males had a slight preference to play in dry sand more than 5-year-old females, but this was moderately significant (*p* = 0.096).

**Figure 3:**
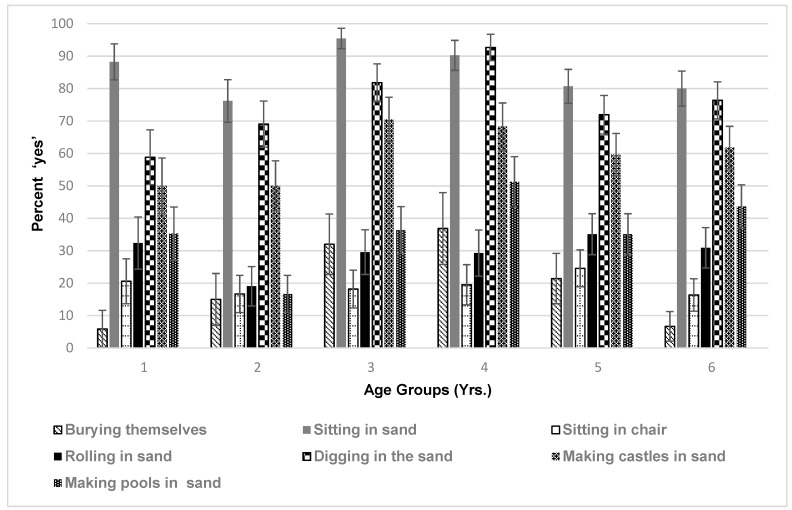
Percent ‘Yes’ for Being Engaged in Certain Activities. For ‘burying themselves’: significant difference between age groups 1 years and 3 years (*p* = 0.018), 1 years and 4 years (*p* = 0.014), and between 6 years and 3 years (*p* = 0.015), and 6 years and 4 years (*p* = 0.012). For ‘sitting in the sand’: significant difference between 2 and 3 year-olds (*p* = 0.008), a moderately significant difference between 2- and 4-year-olds (*p* = 0.081), a significant difference between 3- and 5-year-olds (*p* = 0.016) and 3- and 6-year-olds (*p* = 0.014). For ‘rolling in sand’: moderately significant difference between age group 2 years, and 5 years (*p* = 0.067). For ‘digging in the sand’: significant difference between age group 1 year and 3 years (*p* = 0.025), 1-year-olds and 4-year-olds (*p* < 0.001) and a moderately significant difference between 1-year-olds and 6-year-olds (*p* = 0.086). There was also a significant difference between 2-year-olds and 4-year-olds (*p* = 0.004), 4-year-olds and 5-year-olds (*p* = 0.004), and 4-year-olds and 6-year-olds (*p* = 0.021). For ‘making castles’: moderately significant difference between children aged 1 year and 3 years (*p* = 0.63), a significant difference between children aged 2 years and 3 years (*p* = 0.048) and moderately significant difference between 2- and 4-year-olds (*p* = 0.085). For ‘making pools’ in sand, there was a moderately significant difference between 1- and 2-year-olds (*p* = 0.063). There was also a significant difference between children aged 2 years and 3 years (*p* = 0.034), children aged 2 years and 4 years (*p* = 0.0001), children aged 2 years and 5 years (*p* = 0.031), and children aged 2 years and 6 years (*p* = 0.002).

Researchers were also interested in how children play in the sand, to evaluate the level of interaction with potentially contaminated sand (Figure 3). There are some notable difference in the age groups, and what they prefer. The 1-year-olds and 6-year-olds did not bury themselves as much as the other children, in particular compared to the 2- and 3-year-olds. The 1-year-old also did not dig in the sand as much as the other children, while the 3-year-old seemed to prefer sitting in the sand the most. All children enjoyed making castles in the sand, between 50 to 70 percent of the time on average across age groups, with the younger children spending less time in this activity due to time spent in the arms of parents or guardians.

There were no significant differences between males and females, for burying themselves in the sand and sitting in the sand. With reference to sitting in a chair, 3-year-old females preferred to sit in the chair more than 3-year-old males (*p* = 0.034) with 5-year-old males moderately significantly preferring to sit in the chair more than 5-year-old females (*p* = 0.076). Looking at rolling in the sand, 5-year-old males preferred to roll in the sand more than 5-year-old females (*p* = 0.031). Regarding digging in the sand, there was a significant difference with 3-year-old females preferring to dig in the sand more than males (*p* = 0.002). With reference to making castles, 3-year-old females preferred to make castles more than 3-year-old males (*p* = 0.011). In addition, 5-year-old males preferred to make castles more than 5-year-old females (*p* = 0.008). Regarding making pools, there was no significant differences between genders.

**Figure 4:**
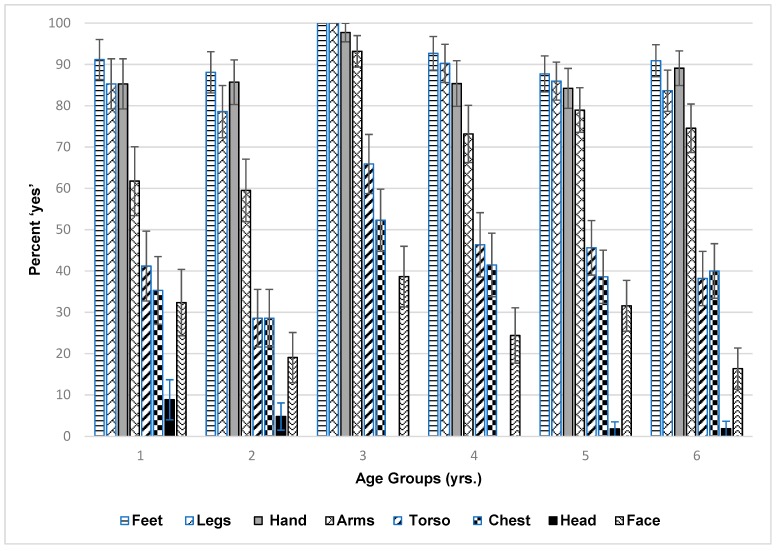
Child body part in contact with sand by age group. Feet: moderately significant difference between age group 1 year and 3 years (*p* = 0.070), significant difference between age group 2 years and 3 years (*p* = 0.017), moderately significant difference between 3- and 4-year-olds (*p* = 0.072), significant difference between 3- and 5-year-olds, and 3- and 6-year-olds. Legs: significant difference between age group 1 year and 3 years (*p* = 0.016), and between age groups 2 years and 3 years (*p* = 0.001), 3 years and 4 years (*p* = 0.036), 3 years and 5 years (*p* = 0.002), and 3 years and 6 years (*p* = 0.001). For hands: moderately significant difference between 1- and 3-year-olds (*p* = 0.055), a significant difference between 2- and 3-year-olds (*p* = 0.040), 3- and 4-year-olds (*p* = 0.038), 3- and 5-year-olds (*p* = 0.011), and a moderately significant difference between 3- and 6-year-olds (*p* = 0.070). For arms: significant difference between age group 1 year and 3 years (*p* = 0.001), a moderately significant difference between 1 year and 5 years (*p* = 0.084), a significant difference between age group 2 years and 3 years (*p* < 0.001), 2- and 5-year-olds (*p* = 0.037), 3- and 4-year-olds (*p* = 0.011), -, and 5-year-olds (*p* = 0.031), and 3- and 6-year-olds (*p* = 0.008). For torso: significant difference between age groups 1 year and 3 years (*p* = 0.026), a significant difference between age group 2 years and 3 years (*p* = 0.0001), a moderately significant difference between 2- and 4-year-olds (*p* = 0.090), a moderately significant difference between age groups 2- and 5-year-olds (*p* = 0.076), a moderately significant difference between 3- and 4 year-olds (*p* = 0.065), a significant difference between 3- and 5-year-olds (*p* = 0.037), and between 3- and 6 year-olds (*p* = 0.004). For chest: a significant difference between age groups 2- and 3-year-olds (*p* = 0.021). For head: a moderately significant difference between 1- and 3-year-olds and 1- and 4-year-olds only (*p* = 0.070). For face: a moderately significant difference between age groups 1- and 6-year-olds (*p* = 0.091), a significant difference between 2- and 3-year-olds (*p* = 0.040), and 3- and 6-year-olds (*p* = 0.012), and a moderately significant difference between age groups 5 years and 6 years (*p* = 0.055) for face in contact with sand.

Researchers were interested in the level of contact of children body parts with sand for feet, legs, hands, arms, torso, chest, and head (Figure 4). In general, feet, legs, arms and hands spent the most time in contact with the sand for all children, the greatest being the feet. However, the younger age groups had their legs in contact with sand the least. The 3-year-olds had the greatest contact for their arms, torso, and chest. Very little contact was reported for the face and head for all children. There is likely to be a relationship between what children do in the sand (Figure 3) and their body parts in contact with the sand (Figure 4). Building castles, digging, and rolling in the sand should bring more of the body parts, such as the head in contact with the sand. However, obvious consistent associations were not apparent. For example, the age group that liked to roll the most in the sand were the 5-year-olds, however their chest was not in contact more than other ages. In a separate analysis of females versus males for body parts in contact with sand for each age group, there was only a statistically significant difference for 5-year-olds, where males had their face in contact with the sand more than females (*p* = 0.025).

**Figure 5:**
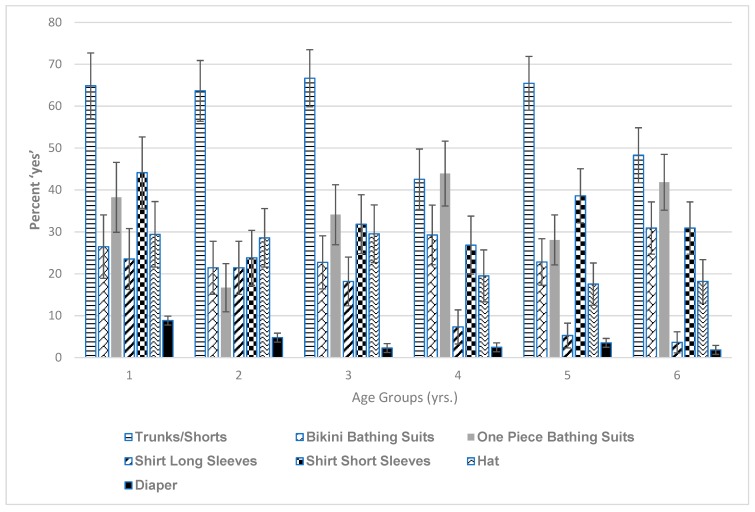
Child clothing preferences by age group. For trunks: a significant difference between 1- and 4-year-olds (*p* = 0.037), 2- and 4-year-olds (*p* = 0.040) and 3- and 4-year-olds (*p* = 0.015), a moderately significant difference between 3- and 6-year-olds (*p* = 0.052), a significant difference between 4- and 5-year-olds (*p* = 0.018) and a moderately significant difference between 5- and 6-year-olds (*p* = 0.061). For one piece bathing suits: significant difference between age groups 1 year and 2 years (*p* = 0.034), a moderately significant difference between 2- and 3-year-olds (*p* = 0.058), 2- and 4 year-olds (*p* = 0.005), and 2- and 6-year-olds (*p* = 0.004). For long sleeved shirts: a significant difference between 1 year and 5 years (*p* = 0.020), 1- and 6-year-olds (*p* = 0.010), 2- and 5-year-olds (*p* = 0.021), and 2- and 6-year-olds (*p* = 0.009). In addition, there was a significant difference between 3- and 5-year-olds (*p* = 0.048) and 3- and 6-year-olds (*p* = 0.022) and a moderately significant difference between children aged 1 year and 4 years (*p* = 0.052), and 2- and 4-year-olds (*p* =0.061). For short sleeved shirts: a moderately significant difference between children aged 1 year and 2 years (*p* = 0.059).

To further evaluate the body parts exposed to sand, the types of clothing typically worn by children when visiting the beach were also evaluated by age group (Figure 5) and by gender. In the overall analysis, trunks were the most common type of clothing worn across all age groups, although least preferred by 4- and 6-year-olds. Naturally, the younger children were more likely to wear diapers and the data indicated this, but there were no statistically significant differences in the age groups regarding wearing diapers given the low numbers in this category. In terms of separating the clothing analysis by gender; overall, males wore trunks/shorts more than females and this was significant across all age groups: 1-year-olds, (*p* = 0.013), 2-year-olds (*p* = <0.001), 3-year-olds (*p* < 0.001), 4-year-olds (*p* < 0.001), 5-year-olds (*p* < 0.001), and 6-year-olds (*p* < 0.001). As expected, females wore bikinis significantly (over 85% for age groups) more than males for all age groups *p* < 0.001 for all age groups). For one-piece bathing suits, females also wore these significantly more than males (over 84% across age groups): 1-year-olds (*p* = 0.019), 2-year-olds (*p* = 0.002), 3-year-olds (*p* = 0.002), and for 4-, 5-, and 6-year-olds (*p* = <0.001). For short sleeves, 4-year-old males wore these more than 4-year-old females, where this was moderately significant (*p* = 0.077). This was also true for 6-year-old males compared to 6-year-old females (*p* = 0.066). With reference to hats, there were moderately significant findings; 4-year-old males wore hats more than 4-year old-females (*p* = 0.099) and 6-year-old males more than 6-year-old females (*p* = 0.073).

#### 3.2.3. Exposure Subsection 3

For items that children put in their mouth, the analysis was also completed by comparing age groups. There was a moderately significant difference between 1- and 2-year-olds (*p* = 0.083) and 1- and 3-year-olds (*p* = 0.081), where 40.8% of 1-year-olds like to put items in their mouth (2-year-olds were 22.7%, and 3-year-olds were 22.9%). There was a statistically significant difference between 1- and 4-year-olds (*p* < 0.001), 1- and 5-year-olds (*p* = 0.001), and 1- and 6-year-olds (*p* = 0.002) for putting items in the mouth (11.0% and 12.1% of 5- and 6-year-olds, respectively like to put items in their mouth). There were no significant differences between genders across age groups for preference for putting an item in the mouth. From the survey items identified that children could put in the mouth include, seashells, sand, seaweed, or toys. Parents also responded about observing sand on the bodies of children. There were no statistically significant differences between the age groups or between the genders, where across age groups there was a 70–84% positive response about observing sand on the body.

Parents overwhelmingly said that they do bring snacks to the beach (83.2%), where 11.5% said sometimes (NR was 4.8%). When it comes to buying snacks, it was almost split into thirds between ‘yes’ (32.3%), ‘no’ (33.0%), and ‘sometimes’ (34.8%) (however, NR was high at 30.3%). This may be a function of which beach they visit typically, the availability of food, the length of time they stay and the cost of food. This question was of interest to the research team and might affect more hand to mouth activities while on the beach increasing ingestion exposure. However, this might ultimately be affected by the hygiene activities of parent and child (e.g., washing hands before eating). Some of the snacks respondents described as bringing included water, juice, fruits, sandwiches, chips, nuts, smoothies, pizza, berries, apple sauce, jelly, and yogurt. Sunscreen use was an important aspect of this project because it is hypothesized that sand would stick more readily to skin where sunscreen is present. Of all respondents, 86.6% reported using sunscreen on their children while they are at the beach and 68.9% reported a re-application (NR 20.5%). The use of sunscreen highlights parent’s emphasis on protecting children from the harmful effects of sunlight, as is recommended by health professionals [27].

Children’s hygiene habits may also affect the transfer of OSCs from hands to mouth or duration on skin for dermal uptake and absorption. According to the surveys (Table 1), 56.5% of children wash their hands before they eat although most people do not bring soap to the beach (82.5%). Those in Texas responded that they wash their hands more before eating than those Miami (37% to 63%: *p* < 0.001). The survey however did not ask whether parents brought wipes, or another means of cleaning hands at the beach. About 62.5% of children use the shower to rinse the sand off themselves while 68.1% use the foot rinse. About 74% of parents answered ‘yes’ or ‘sometimes’ to taking their children shopping or to a restaurant after the beach, while 26.0% did not or were uncertain. Responses also showed that 74.1% of parents or guardians had their children bathe immediately after getting home, 15.8% responded ‘bathing within an hour’, 6.5% ‘within a couple hours’, and the other 10.1% either ‘later that night’, ‘the next day’, or ‘uncertain’.

Respondents also answered for each one of their children, whether they like to bathe themselves, need help, or both. Responses were analyzed by age group and there were significant differences in the responses of the younger children compared to the older children. Younger children needed more help than the older children. The responses ranged from 87.9% needing help to 38.8% for 1-year-olds to 6-year-olds (*p* values from 0.025 to <0.001 for significant differences in age groups). Likewise, for bathing themselves, older children preferred to bathe themselves. The responses ranged from 3.0% to 24.5% for the 1-year-olds to the 6-year-olds (*p*-values from 0.014 to <0.001 for significant difference in age groups). For the combined preference of both bathing themselves and getting help, across age groups that percent ranged 9.1–36.7% from 1-year-olds to 6-year-olds with some significant differences between age groups.

### 3.3. Risk Perception Results

The risk perception section of the survey focused on parent’s perceptions of risks associated with the beach, their preferences on receiving alerts, and knowledge of tarballs. Most subjects believed that it was possible to get sick after visiting the beach (‘yes’ at 47.2% and ‘maybe’ at 22.6%), but most said their children have never been sick from the beach (‘sick’ 8.5%, ‘not sick’ 80.5%, and ‘maybe sick’ at 4.0%, ‘uncertain’ was 7.0%). For the few respondents that said their children got sick, 61.2% responded at the Miami beaches while 38.2% responded at the Texas beaches, however the data was not sufficient to confirm significance (see Supplementary Table S3). For the 38 respondents who did describe an actual illness, they reported events such as colds, hives, fevers, rashes, coughs, earaches, muscle cramp, excess sun exposure, and diarrhea. For those who thought it was possible to get sick after visiting the beach they also listed things such as sunstroke, rashes, allergic reactions, stings, gastrointestinal problems, and cold or ear infections (only 21 responded). The majority of identified health outcomes (e.g., colds, ear infections, diarrhea) appear to be related to more common acute viral and bacterial infections on beaches as opposed to outcomes from exposures to OCSs that would occur more likely from long-term exposures to low chemical concentrations. A number of studies have found illnesses following beach swimming and linked to presence of indicator bacteria levels, where symptoms include diarrhea and rashes for example [28,29], although sometimes the association was limited or not found [30,31]. Deflorio-Barker et al. [32] examined 12 studies to estimate the ingestion of water, sand, or algae and potential relationship to gastrointestinal illnesses. Young children aged 4–12 swallowed a greater amount of water for example compared to other age groups, increasing their potential exposures [32].

Along with the potential for getting sick, perceptions of beach safety was also explored. Results showed that 82.2% of parents typically saw signs and postings on the beach area and that 88.9% paid attention to those signs and postings. There was no significant difference between beaches for the response to these questions on signs and postings. Results also showed that 80.8% of parents or guardians have heard warnings about beach safety and beach closings, while 10.5% said ‘no’ and the remaining percentage were either ‘maybe’ or ‘uncertain’. In terms of where the warnings were observed: 66.7% said they learned about them from TV news, 53.2% from postings on the beach, 42.6% from social media, 33.0% from the radio, 28.8% by word of mouth, 9.3% from newspapers, and 9.0% each from schools and cell phone app notifications (no response rate was 16.8%). With that, most participants prefer to be notified about beach closings from TV news (58.3%), postings on the beach (51.3%), and social media (48.0%) (NR 1%). Other ideas recommended by participants for where to get notices in general and about beach closings included: city websites, email, Instagram, weather application, and phone text. Most (86.5%) participants noted that when they did get notices, they are easy to understand (NR 3.5%).

Given the changes in the growth and dependence on social media over the last decade, we evaluated differences in preference for warnings by age. A binary logistic regression to determine preference for receiving warnings by age indicated that participants under 30 were less likely to prefer methods such as radio, TV, postings on the beach, schools, newspapers, social media, or word of mouth, where postings on the beach was significant (OR 0.577; 95% CI 0.358–0.926; *p* = 0.023). Participants under 30 were significantly likely to prefer to receive warning via their cellphones (OR 1.889; 95% CI 1.172–3.041; *p* = 0.009).

Survey results showed that 66.3% of respondents did not have concerns when they visited the beach, while 28.1% did (NR 2%). About 99 respondents listed some of those concerns as: tide and current, infections, water safety, broken glass, marine life, sun safety, seaweed, playing on rocks, feces, lighting at night, drugs and paraphernalia. Respondents were then asked to rank the concerns for dangers of infections, shark attacks, strong waves, rip tides, hurricanes, and chemical or oil spills on a scale of 1 (least concern) to 10 (most concern). Table 2 shows that infections received the highest ranking of 10 among the most participants (39.7%) while strong waves received the highest ranking of 10 among the least participants (23.4%). The items listed as ‘other’ were indicated as potentially: shards of glass on the beach and pedophiles.

Another safety aspect of the beach is overall appearance of cleanliness, where 88.1% of participants said that they would not let their children play on the beach if it appeared dirty (NR 1.8%). Respondents considered a dirty beach as having the presence of trash (89.1%), some tar or oil (78.2%), seaweed (51.5%), or various other items (2.0%), where some of those other items were noted as glass and needles. Subjects could select more than one response to this question.

Regarding an awareness of oil on the beaches, 93.8% of parents have never observed oil stains on their children after playing on the beach (NR 3.8%). For the few individuals (totaling 8) who responded to how much oil they observed, they mostly commented that that they observed oil on portions of the feet. Most subjects were not familiar with tarballs (55.8%), where 8.2% were either ‘maybe’ or ‘uncertain’ (NR 3.5%). Respondents were then shown a picture of a tarball, which are dark or black/brown pellets or chunks that might be indicative of the presence of oil. Subjects were then asked if they had seen tarballs on the beach within the last year, where 87.6% had not, however 8.8% were uncertain (NR 3.5%). When asked if they had ever seen tarballs, 76.0% responded with a negative answer. A binary logistic regression revealed that those at Texas beaches were more likely to have seen a tarball within the last year compared to those at Miami beaches, based on the ones that did answer the question (OR 1.78; 95% CI 1.00–3.18; *p* = 0.049). Galveston beaches have been noted to have a number of oil spills routinely [33], potentially explaining why respondents at the Galveston beaches in Texas were more familiar with tarballs.

### 3.4. Key Comparison of Demographics, Exposure, and Risk Perceptions by Race

Results show a significant difference in racial makeup between regional beaches. Whites represented 75.4 percent of the total participants in the study, where a greater proportion of the White respondents (60.9% total) were surveyed at the Miami beaches versus at the Texas beaches (39.1% total) (*p* < 0.001).

Those who identified as White were also more likely to have a higher income (OR 1.37 95% CI 1.18–1.59; *p* < 0.001), while those who identified as Black were less likely to have a higher income (OR 0.71: 95% CI 0.59–0.85; *p* < 0.001) (income ranges shown in Table 3). Additionally, a binary logistic regression was performed with race as the dependent variable to determine the likelihood of events of interest. Those identifying as Black were more likely to live further from the beach (OR 1.34; 95% 1.12–1.60; *p* = 0.002). However, no association was found between race and frequency at beach, distance from beach, age, and hours worked in their primary job.

Regarding preference for receiving warnings on the beach, Whites were more likely to prefer social media and cell phones, with cell phones being statistically significant (OR 1.947; 95% CI 1.219–3.110; *p* = 0.005). Whites were less likely to prefer radio, TV, postings on the beach, schools, newspapers or word of mouth with preference for TV being statistically significant (OR 0.521; 95% CI 0.334–0.809; *p* = 0.004) and postings being moderately significant (OR 0.675; 95% CI 0.442–1.030; *p* = 0.069).

Regarding preference for receiving warning on the beach, Blacks were more likely to prefer radio, TV, postings, schools, newspapers, and word of mouth. Radio was moderately significant (OR 1.710; 95% CI 0.987–2.961; *p* = 0.056), while postings (OR 2.033; 95% CI 1.173–3.524; *p* = 0.011), TV (OR 1.852; 95% CI 1.050–3.268; *p* = 0.033), and newspapers (OR 2.228; 95% CI 1.181–4.203; *p* = 0.013) were statistically significant. Finally, Blacks did not prefer social media or cell phones, with cell phones being statistically significant (OR 0.504; 95% CI 0.272–0.935; *p* = 0.030).

In a binary logistic regression of Blacks versus Non-Blacks exploring concerns when visiting the beach, Blacks were more likely to be concerned about infections, hurricanes, sand storms, and chemical oil spills, and less likely to be worried about strong waves and rip tides, where only the comparison for rip tides was statistically significant (OR 0.964; 95% CI 0.88–1.06; *p* = 0.045). For White versus Non-Whites, Whites were less likely to be concerned about all categories of dangers, where shark attacks were statistically significant (OR 0.912; 95% CI 0.851–0.978; *p* = 0.010) and hurricanes and strong storms were moderately significant (OR 0.073; 95% CI 0.877–1.01; *p* = 0.073). Increased perception of risk is consistent with other studies that have found that among Black communities there is a greater intergenerational transmission of the risks associated with coastal and marine visitation [34].

## 4. Conclusions

This project was the first of its kind to collect more detailed activity patterns on families and their young children in the beach environment. The majority of respondents were White, although a higher percentage of Blacks completed the survey in Texas. The highest income category of $50,000 to $100,000 falls within the national average range of $57,652 [35]. However, if the survey allowed for a dollar amount entry, a better comparison to the national average could have been determined to see if higher incomes within that range affects the ability to live near the beach or visit the beach. Most took their children to the beach once a year or once a month, although regionally there were differences, where individuals in Miami frequented the beach more. Parents and guardians also tend to stay longer when they take their children. Most drive to the beach, which was common in other studies (e.g., [23]). Like authors Dwight et al., [22], this study also found that most families go to the beach during the summer (69.8%) and that most participants said they go to the beach based on the weather being pleasant, and whether the sun was up and their work or children’s school schedule allowed.

Our ability to compare the exposure-related activities to other studies is difficult given the lack of research in the literature, where this study collected unique information related to individual play activities and hygiene practices. Children’s exposures may be influenced by some of the following factors: what children wear, how much time is spent on the beach, how often they wash their hands, and where they spend time while at the beach. Overall, the initial data analysis showed high variability across how children play and interact in the beach environment by age, although we see less variation in behavior by sex. In addition, parents and children may not be consistent with their hygiene practices, where we surprisingly observed differences in hygiene practices by beach region (i.e., Texas vs. Miami). We also find perceptions of risk vary greatly among families. In particular, risk perception and preference for messaging is seen to vary by race in this study. We also observed some socio-demographic differences by race (e.g., blacks had a lower income and lived further from the beach), where the association between income and race is a common finding. Given the lack of data on children behaviors in the beach environment, researchers are encouraged to survey other families in other beach regions for comparison. The Heaney et al. [18] study which evaluated children’s behaviors in the sand at lake beaches offers some comparison, although they have broad age groups of 0–4 and 5–10 years of age. On average, 20% of our 1- to 6-year-olds liked to bury themselves in the sand and 75.0% like to dig in the sand, whereas 17.0% of the 0- to 4-year-olds in the comparison study like to bury themselves and 15.0% like to dig in the sand. The time spent digging in our study is substantially larger and may be affected by design of survey and or how people behave differently on freshwater lake versus marine coastal beaches.

There were some limitations with the project and with some of its methodologies. For example, the survey method relies on the recall ability of the respondent to report their activities and the activities of their children. Parents or guardians may remember their general behaviors such as how they get to the beach and how often they typically visit; however, some errors may occur in how they relate their children’s behaviors such as how much time they typically spend playing in areas of the beach (i.e., intertidal or water). Greater error may occur for a respondent who had to recall detailed activities (e.g., rolling in sand) for multiple children. Because the survey was delivered in the beach environment, there was likely to be a bias toward families and children who visit the beach more than average families across the country. Other errors may have occurred due to collection of the survey in an open and more active environment where distractions are possible. There may also have been difficulties in the interpretation of questions by respondents (e.g., did parents understand what the ‘intertidal area’ meant).

Additional objectives of the overall Beaches project will address the fate and transport of chemical constituents associated with oil spills to evaluate potential exposure risks in children using impacted beaches. Survey data (e.g., time spent at the beach daily and yearly) reported here, videotaping data (e.g., contact with seaweed, sand and time spent in intertidal zone), and sand adherence measured will be combined with fate and transport measured media concentrations to estimate exposure and dose for ingestion, inhalation, and dermal health risk for young children. A previous publication from this group, performs a preliminary risk assessment for beach exposure using existing databases for children’s exposure parameters as defaults, but calls on a need for refined exposure parameters for this population and environment [36,37]. This survey data offers refinement in child visits and time spent at beaches and offers considerable data on contact activities for children 1–6 years of age.

As a secondary objective from the survey collection and analysis, risk perception questions from the survey will later be used to improve safety messaging for parents based on their existing knowledge base and how they prefer to receive information. Appropriate messaging can also be used to influence their behaviors and current perception of risks to oil, bacteria, and other environmental hazards found at beach areas. In addition, coastal managers may find the risk perception (e.g., avenues for messaging) and general behaviors (e.g., frequency of visits to the beach) useful for planning and maintenance of beach areas.

In conclusion, the immense data on children activities and behaviors presented here, which is the main focus of the study, may prove useful for those evaluating children exposures to a variety of contaminants including those of bacterial [38] and chemical origin [36].

## Figures and Tables

**Table 1:** Hygiene Practices.

	**% Yes**	**% No**	**% Sometimes**	**% Uncertain**		**NR ^a^ %**
Stay longer at beach if bathroom available	47.8	31.0	11.1	10.1		3.3
Children wash hands before eating	56.5	23.2	12.5	1.8		21.3
Go to other places after the beach	30	24.8	43.9	1.3		3.3
Use of shower to rinse yourself	63.4	22.3	12.5	1.8		3.8
Use of shower to rinse children	62.5	24.2	12.8	0.6		10.0
Use foot rinse for children	68.1	20	10	1.9		10.0
Take soap to beach	9.6	82.5	7.1	0.8		1.3
	**Every** **Hour**	**Couple Hours**	**Before** **Meals**	**Before Going Home**	**Never/Uncertain**	
How often is soap and water applied to children’s hands	3.4	13.6	55.9	47.5	5.1/5.1	85.3
How often is soap and water applied to your hands	5.1	12.8	48.7	48.7	5.1/7.7	90.3
Timing of children have a bath or shower upon arrival at home	Right away: 74.1%	Within 1 h: 15.8%	Within couple h: 6.5%	Later that night: 3.0%	Next day/uncertain: 0/0.5%	0.5

^a^ NR: No response.

**Table 2:** Potential Dangers at the Beach that were Ranked as ‘Most Concerning’.

	Mean Percent in Ranking ^#^										NR ^a^ %
Danger/ranking ^#^	10	9	8	7	6	5	4	3	2	1	
Infections	39.7	6.1	7.4	4.7	4.7	8.1	3.0	6.4	5.7	14.1	25.8
Shark attacks	31.8	5.8	3.9	5.1	6.8	5.8	5.1	6.8	15.8	13.2	22.3
Strong waves	23.4	7.5	12.3	9.4	5.2	13	6.2	8.1	5.8	9.1	23.0
Rip tides	29.2	8.2	10.5	7.2	7.5	8.5	6.9	5.2	7.2	9.5	23.8
Hurricanes or strong storms	35.4	5.7	4.7	5.7	6.4	5.7	2.7	6.7	6.7	20.2	25.8
Chemical or oil spill	34.7	8.1	5.7	4.0	4.0	9.1	3.4	5.1	9.8	16.2	25.8
Other	35.7	7.1	0.0	7.1	0	21.4	7.1	7.1	0	14.3	96.5

^#^ Ranking order for dangers. ^a^ NR: No response.

**Table 3:** Demographics by Race.

Characteristics	Overall (*N* = 359)	White (*N* = 271)	Black African American (*N* = 66)
Age			
Mean (SE)	37.95 (0.58)	37.51 (0.71)	39.81 (1.47)
Median (IQR)	37 (30–44)	36 (29–43)	39 (32.5–41)
Sex			
Male	28.2%	28.8%	25.86%
Female	71.8%	71.2%	74.14%
Income			
Mean Range	30,001–100,000	30,001–100,000	20,001–50,000
Median Range	50,001–100,000	30,001–100,000	30,001–50,000
Hours Worked in Primary Job (SE)	38.30 (0.78)	37.96 (1.04)	40.14 (1.58)
Mean Range Distance Live From Beach	10–30	5–20	10–30
Mean Range Frequency to Beach	Once a week–Once a month	Once a week–Once a month	Once a week–Once a month

## Supplementary Materials

The following are available online at https://www.mdpi.com/1660-4601/16/15/2783/s1, Table S1: Demographics of Respondents; Table S2: Beach Dynamics and Profile of Children; Table S3: Risk Perceptions. IRB approvals letters are also found in Supplement.
