# Supplementary material for: Children Exposure-Related Behavior Patterns and Risk Perception Associated with Recreational Beach Use
Source: Int J Environ Res Public Health. 2019 Aug 3;16(15):2783. doi: 10.3390/ijerph16152783 (PMC6696461; doi:10.3390/ijerph16152783)
Supplement: Supplementary file 1 [file ijerph-16-02783-s001.pdf]

International Journal of
Environmental Research and Public Health

MDPI

Children Exposure-Related Behavior Patterns and Risk Perception Associated with Recreational Beach Use

Supplementary Material

Table S1. Demographics of Respondents.

**Age**	Mean: 37.95		Min: 17	Max: 73			NR*: 3.3 %
**Sex**	Female: 71.8%	Male: 28.2%					NR: 16.8%
**Hispanic Descent**	No: 54.4%	Yes: 46.6%					NR: 2.5 %
**Race**	White: 75.5%	Black: 18.4%	Other: 6.1%				NR: 10.3%
**Household Income**	<$12,000 8.6%	<$20,000 5.0%	<30,000 11.8%	<50,000 16.2%	<100,000 25.4%	>100,000 19.9%	NR: 13.1%
**Primary Occupation**	e.g., student, housewife, nurse, electrician, cook, law enforcement, physical therapist, nanny, CAD designer, engineer, plumber, realtor						NR: 6.8%
**Hours worked in Primary Occupation**	Mean: 38.30	SE: 0.78	Min: 3	Max: 74			NR: 46.8%
**Primary Occupation of Spouse**	e.g., seamstress, pilot, certified public accountant, barber, engineer, stock broker, mechanic, personal trainer, apartment manager, social worker						NR: 39.8%
**Hours worked in Primary Occupation of Spouse**	Mean: 42.79	SE: 0.81	Min: 12	Max: 84			NR: 60.8

*NR: No Response Rate .

Table S2. Beach Dynamics and Profile of Children.

**No of children (<18) that go to the beach who live in home of respondent**	Mean: 1.85	SE: 0.056	Min: 0	Max: 8		NR[a]%: 7.3
**No of children respondent typically takes to the beach**	Mean: 2.16	SE: 2.16	Min: 0	Max: 14		NR%: 8.3
**Relation of children to adult respondent**	Parent: 79.8%	Grandparent: 10.5%	Guardian: 2.0	Other: 7.8%		NR%: 0
**Distance staying/living from beach in miles**	< 5: 26.8%	<10: 13.2%	<20: 17.1%	<30: 12.2%	>30: 30.6	NR%: 3.8
**Method to get to beach**	Drive/car: 91.3%	Walk: 9.9%	Bus: 0.5%	Other: 1.0%		NR%: 2

[a]NR: Non Response Rate.

Table S3. Risk Perceptions.

Questions	%Yes	%No	%Maybe	%Uncertain	NR[a]%
**Have gotten sick after visiting beach?**	8.5	80.5	4.0	7.0	0.3
**Possible to get sick after visiting beach?**	47.2	15.8	22.6	14.3	0.5
**Do you see signs and posting on the beach area?**	82.2	10.5	4.8	2.5	0.3
**Do you pay attention to signs and postings?**	88.9	3.3	6.6	1.3	1.3

[a]NR: No response.
